# A New Insight on the “S” Shape Pattern of Soft Faults in Time-Domain Reflectometry

**DOI:** 10.3390/s23249867

**Published:** 2023-12-16

**Authors:** Florent Loete

**Affiliations:** GeePs, Group of Electrical Engineering Paris, CNRS, CentraleSupélec, Université Paris-Saclay, 3 & 11 Rue Joliot-Curie, 91192 Gif-sur-Yvette, France; florent.loete@centralesupelec.fr

**Keywords:** soft defect modeling, reflectometry, electrical harnesses diagnosis

## Abstract

This paper proposes a new interpretation of the commonly observed, very specific, time-domain response associated with a soft defect in an electrical line under test using time-domain reflectometry. The reflectometry reveals the nature of a defect by analyzing the reflections undergone by an injected pulse at the impedance discontinuities present on the line. The faulty section considered in this work is modeled as a local modification of the characteristic impedance. Using the developed model, we explain how, depending on the physical and electrical characteristics of the faulty section, the associated signature yields a very specific “S”-shaped pattern. The influence of the probing signal is also investigated. Finally, it is shown that the amplitude of the reflected signal cannot be interpreted straightforwardly as a mirror of the severity of the defect and that consequently, small echoes can mask more significant defects.

## 1. Introduction

Transmission lines are subject to premature aging because of the severe environmental conditions they are placed in. Mechanical, chemical, or thermal stress may result in accidental alteration in the geometrical and electrical nominal properties of the cable [1]. Consequently, the cable may be locally damaged, and its ability to safely transmit signals between systems is jeopardized. Hard faults (open and short circuits) result in an immediate malfunctioning of the affected systems. On the contrary, soft faults, arising from partial damage to the line, may be present without being noticed for a while. They usually evolve toward harder defects that can lead to the total malfunction of the system. But one must not forget that they have also been proven to be potentially catastrophic since an insulation defect may result, for example, in arcing [2]. The potential severity of soft defects should therefore not be minimized.

During the past decade, time-domain reflectometry (TDR) has proven to be an effective tool for the detection and localization of defects on an electrical line [3,4,5]. Hard faults are easily detected, but soft fault detection remains a difficult task since their associated TDR signature is weak [6]. Visual inspection is time-consuming and the cost is elevated, especially if there is no clue about the existence of a defect and its whereabouts. Since the number and length of electrical harnesses in modern systems have dramatically increased, there is consequently a great interest in the development of suitable methods for the detection and characterization of soft defects.

The TDR diagnosis method consists of injecting a high-frequency electrical test signal such as a pulse on a transmission line, where it is reflected at any impedance discontinuity. The location and impedance of the defects can be easily deduced from the measurement of the reflection coefficient of the delayed echoes coming back at the injection point. For hard faults, the measured echo is a scaled version of the injected test pulse. For soft ones, the problem is much more complicated since they are usually characterized by small, spatially localized impedance changes [7]. The resulting TDR signature is often very weak, hidden in the noise [8,9,10,11,12]. The defect is all the more difficult to detect when dealing with a complex electrical harness since its weak signature is mixed with many other ones [13,14]. Consequently, the defect can go unnoticed until it has reached a state where it is already potentially critical. Previous works have also shown that the bandwidth of the testing signal has an influence on the ability of the TDR to detect soft defects with regard to its spatial extension [15]. In this paper, we propose a new derivation that gives a new insight into and a better understanding of the “S” shape signature that is often observed experimentally. It is shown that the frequency characteristics of the test signal as well as the spatial extension and the electrical properties of the defect will influence the shape and magnitude of the measured signature. Consequently, we show that relying on the magnitude of the “S” shape signature for determining the severity of the defect can be greatly misleading.

Experimental results validating the derivation presented in the first section are also presented using an original microstrip setup allowing the realization of well-controlled impedance mismatches.

## 2. Modeling of TDR Patterns in the Presence of Soft Defects

**Figure 1:**
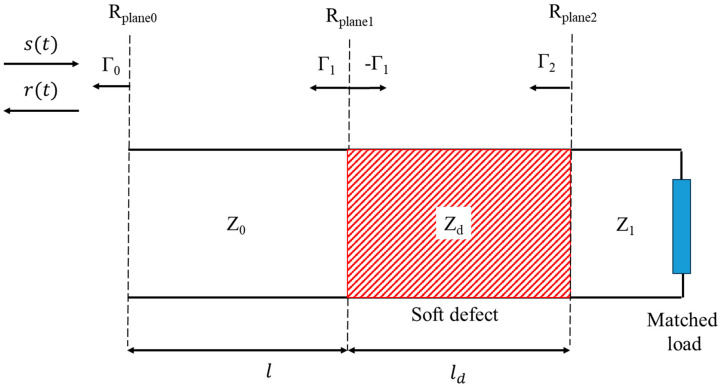
Schematic of a soft defect with a characteristic impedance *Z_d_* inserted on a transmission line. The signal injected endures multiple reflections at the defect interfaces.

In this section, we consider a soft defect inserted on a transmission line of characteristic impedances $Z_{0}$ and $Z_{1}$, respectively, before and after the defect, as shown in Figure 1.

The reference planes R_plane1_ and R_plane2_, defining the defective region, are positioned, respectively, at the distances l and $l+l_{d}$ from the injection point located at the reference plane R_plane0_. A vector network analyzer is used to send a signal *s*(*t*) down the line and measure the amplitude of the reflected signal *r*(*t*) coming back at the injection point. The reflection coefficients at the reference planes R_plane1_ and R_plane2_ which are, respectively, the entrance and the exit plane of the defect are given by:

(1) $$\Gamma_{1}=\frac{Z_{d}-Z_{0}}{Z_{0}+Z_{d}}\;and\;\Gamma_{2}=\frac{Z_{1}-Z_{d}}{Z_{1}+Z_{d}}$$

**Figure 2:**
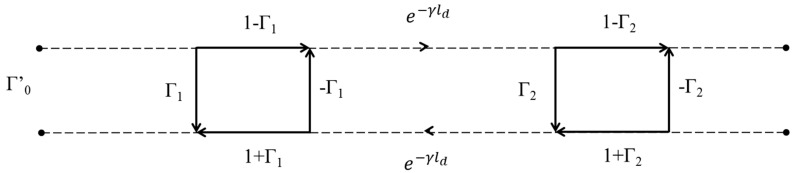
Flow chart of the electrical line including the defect.

Considering the signal flowchart of the defect presented in Figure 2, Mason’s rules [16] allow us to write ${\Gamma^{\prime }}_{0}$, as a function of the scattering parameters of the defect:

(2) $${\Gamma^{\prime }}_{0}=\Gamma_{1}+\frac{(1-\Gamma_{1})(1+\Gamma_{1})\Gamma_{2}e^{-2\gamma l_{d}}}{1-\Gamma_{1}\Gamma_{2}e^{-2\gamma l_{d}}}$$

where $\gamma =(\alpha +i\frac{\omega }{v_{d}})$ is the propagation constant inside the defect and ${\Gamma^{\prime }}_{0}$ is the reflection coefficient measured before the defect, without the portion of the line between the source and the defect.

Using a very simple polynomial division, the following expression can be derived:

(3) $${\Gamma^{\prime }}_{0}=\Gamma_{1}+\left(1-{\Gamma_{1}}^{2}\right)\Gamma_{2}e^{-2\gamma l_{d}}-\left(\Gamma_{1}-{\Gamma_{1}}^{3}\right){\Gamma_{2}}^{2}e^{-4\gamma l_{d}}+\left({\Gamma_{1}}^{2}-{\Gamma_{1}}^{4}\right){\Gamma_{2}}^{3}e^{-6\gamma l_{d}}+\cdots$$

This result clearly shows the contribution of each successive reflection inside the defect as the probing signal bounces back and forth at each reference plane. The first term corresponds to the reflection at the entrance of the defect while the second one accounts for the reflection at the exit of the defect that has gone through the plane R_plane1_ two times. The third contribution corresponds to the echo that bounced two times at R_plane2_ before coming back to the injection point. Going further in the polynomial division would yield many other reflection paths as the signal undergoes multiple reflections. Equation (3) can be easily generalized as follows:

(4) $$\Gamma_{0}=\Gamma_{1}+\sum_{n=1}^{\infty }{\left(-1\right)}^{n-1}\left({\Gamma_{1}}^{n-1}-{\Gamma_{1}}^{n+1}\right){\Gamma_{2}}^{n}e^{-2n\gamma l_{d}}$$

where *n* is the index for the successive echoes inside the defect.

Since in practice, the measurement spectrum is limited to a frequency band or several well-chosen frequencies [5], the function *M*(*ω*) is introduced and the impulse response h(t) of the line in the time domain is obtained by computing the inverse Fourier transform of $\Gamma_{0}\left(\omega \right)$, as follows:

(5) $$h\left(t\right)=\frac{1}{2\pi }\int_{-\infty }^{\infty }{M\left(\omega \right)\Gamma }_{0}\left(\omega \right)e^{i\omega t}d\omega$$

Using (3) and (5) one can write:

(6) $$\begin{array}{c} h\left(t\right)=\frac{1}{2\pi }\int_{-\infty }^{\infty }{M\left(\omega \right)\Gamma }_{1}\left(\omega \right)e^{i\omega t}d\omega \\ \;+\frac{1}{2\pi }\int_{-\infty }^{\infty }M\left(\omega \right)\left(1-{\Gamma_{1}\left(\omega \right)}^{2}\right)\Gamma_{2}\left(\omega \right)e^{i\omega t-2\gamma l_{d}}d\omega \\ \;-\frac{1}{2\pi }\int_{-\infty }^{\infty }M\left(\omega \right)\left(\Gamma_{1}\left(\omega \right)-{\Gamma_{1}\left(\omega \right)}^{3}\right){\Gamma_{2}\left(\omega \right)}^{2}e^{i\omega t-4\gamma l_{d}}d\omega \\ \;+\cdots \end{array}$$

Equation (6) clearly emphasizes that the time-domain impulse response to a soft defect on an electrical line consists of the superposition of multiple echoes at each one of its interfaces.

From (6), one can see that the shape of the time-domain signature will be affected by the following physical parameters:

- The properties of the defect itself, through the impedance mismatch introduced on the line;
- The length of the defect;
- The frequency spectrum of the test signal.

Although it is often assumed in the literature that the impedance $Z_{d}$ of a defect does not depend on the frequency, it is not true in the general case since the absorption properties of the material can be frequency dependent.

The frequency spectrum of the test signal is also often considered continuous but in the case of MCTDR [5], portions of the frequency spectrum can be removed for EMC considerations. In the case of a homogeneous defect and homogeneous bandwidth, the following simplifications can be considered:

(7) $$\left\{\begin{array}{cc} \Gamma_{1}\left(\omega \right)=\Gamma_{1} & \forall \omega \\ \Gamma_{2}={-\Gamma }_{1} & \\ M\left(\omega \right)=1 & {-\omega }_{c}<\omega <\omega_{c}\\ M\left(\omega \right)=0 & \left|\omega \right|>\omega_{c} \end{array}\right.$$

Equations (6) and (7) lead to the following simplified normalized impulse response h(t):

(8) $$h\left(t\right)=\Gamma_{1}[sinc\left(\omega_{c}t\right)-\left(1-{\Gamma_{1}}^{2}\right)e^{-2\alpha l_{d}}sinc{(\omega }_{c}\left(t-2t_{d}\right))]+\cdots$$

**Figure 3:**
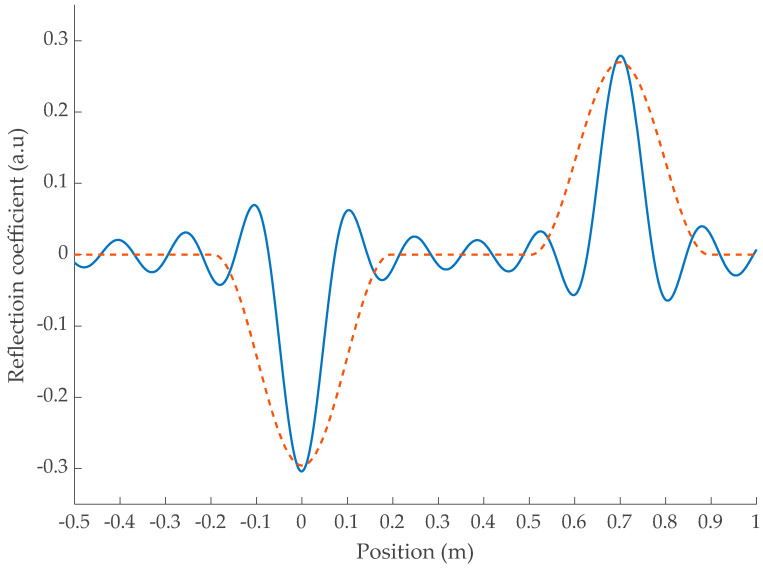
Impulse response of a soft defect with *l_d_* = 0.35 cm and *v_d_* = 0.6*c*, and R_plane1_ = 0 using a rectangular window (**-**) and or a Hann window (**- - **) over a 1102 MHz range. *v_d_* = 0.6*c*.

The typical response of a line including a soft defect is shown in Figure 3. The position on the x-axis is calculated as the product of the speed $v_{d}$ inside the defect and the time *t*. Since a rectangular window introduces a lot of ripples in the time domain, for the rest of the paper we will consider a Hann window for the test signal spectrum $M\left(\omega \right)$. This will conveniently prevent a misinterpretation of the ripples as defects. This window provides a good trade-off between spatial resolution and spectral leakage while only widening the peaks by a factor of 2.

**Figure 4:**
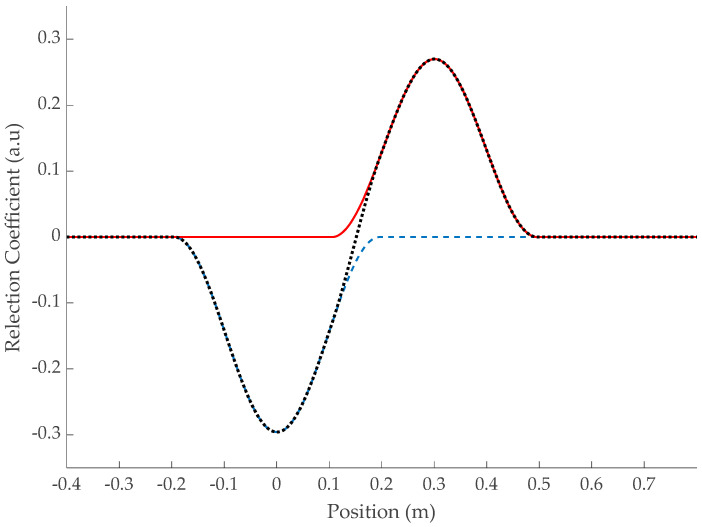
Impulse response of a soft defect with *l_d_* = 0.15 cm and *v_d_* = 0.6*c* (●) using a Hann window over a 1102 MHz range. Separate impulse responses at each interface R_plane1_ (**- -**) and R_plane2_ (**-**).

The multiple reflections inside the defect at R_plane1_ and R_plane2_ emphasized in Equation (8) are easily observed at 0 and $2*l_{d}$, respectively. However, depending on the propagation time $t_{d}$ inside the defect, the echoes may overlap as shown in Figure 4. Since their reflection coefficients are of opposite signs, they may compensate for each other.

The effect starts once their zero-crossings match, but the maximum amplitude of the signature will be prominently affected if the following condition is satisfied:

(9) $$t_{d}\leq \frac{\pi }{\omega_{c}}\;\mathrm{or}\;l_{d}\leq v_{d}\frac{\pi }{\omega_{c}}=l_{dlim}$$

This is a major result since it emphasizes that the apparent magnitude of the signature generated by a soft defect in the time domain must be interpreted very carefully and cannot be a good criterion for estimating its severity, especially if $l_{d}\leq l_{\;dlim}$. As long as the echoes of each interface are well distinct, the amplitude of the peaks allows us to estimate the impedance mismatch. But when (9) applies, and an “S”-shaped soft defect pattern is observed, the reality of the underlying impedance mismatch might be minimized by the overlapping effect of the echoes at each interface.

## 3. Experimental Validation

**Figure 5:**
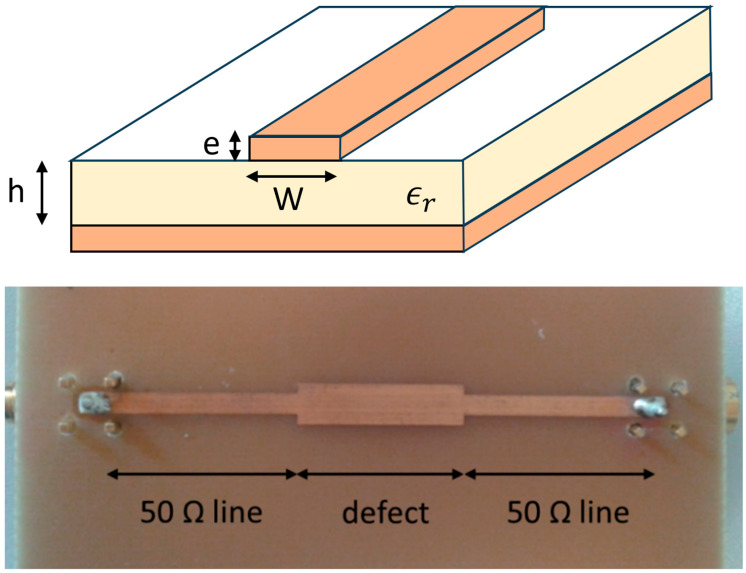
(**Top**) Schematic of a microstrip line. (**Bottom**) Example of a microstrip defect fabricated by locally varying the width of the line.

As it is not easy to create cables with proper impedance-controlled faults, microstrip lines were conveniently used to realize some well-controlled impedance variations along a line. The impedance of the microstrip line presented in Figure 5 can be easily adjusted by changing the width W of the line [17]. This original configuration, though far removed from a real fault case, enabled us to easily produce different defects in order to validate the developments of the first section.

The structure of a microstrip and one of the microstrips we built are shown in Figure 5.

The impedance of the main line was designed to match 50 Ω, while the section in its center could be varied to produce an impedance mismatch of the desired value.

A total of 4 microstrips, including a 25 Ω impedance mismatch with different lengths of 2.5 cm, 5 cm, 12.5 cm, and 35 cm, were realized. The defects were inserted in a 50 Ω terminated RG 58 coaxial line through SMA-mounted connectors. The main line width was 3 mm, the defect width was 7.3 mm, and the FR4 PCB thickness was 1.45 mm.

The impedance of the microstrip lines was calculated using the equation set (10) developed by Wheeler [18]:

(10) w′=w+∆w∆w=e1+1εr2πln10.82eh2+1πwe+1.12Z=42.4εr+1.ln1+4hw′ 4hw′14+8εr11+4hw′214+8εr112+1+1εr2π2

where *w* and *e* are, respectively, the width and the thickness of the microstrip line, *h* is the substrate thickness, and $\varepsilon_{r}$ its relative permittivity.

Using the previous set of equations, and taking into account the precision on the dielectric constant of our PCB material ($4<\varepsilon_{r}<4.3$), the precision on the realized microstrip lines impedances was estimated to be around 5%.

The line was then characterized using an ANRITSU MS2024A vector network analyzer (VNA) over a 1102 MHz range.

**Figure 6:**
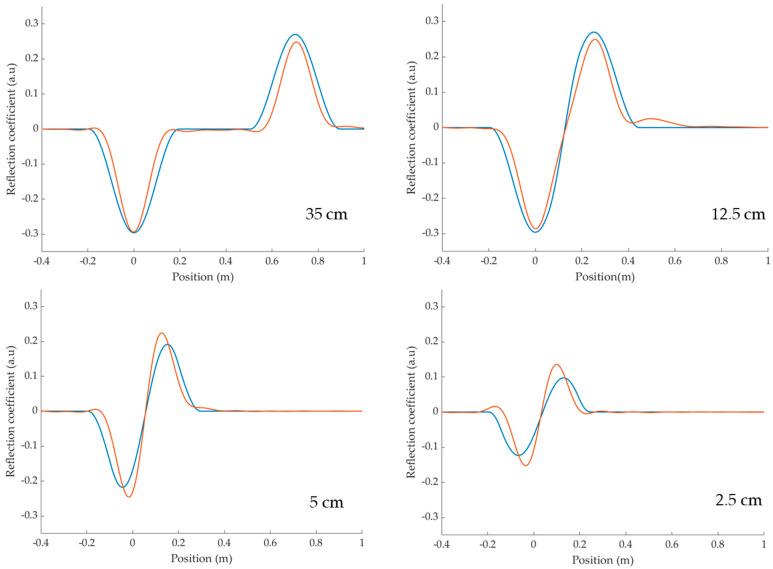
Comparison between the experimental (**-**) and model (**-**) impulse responses of some 25 Ω microstrip soft defects of various lengths inserted on a 50 Ω coaxial line.

**Figure 7:**
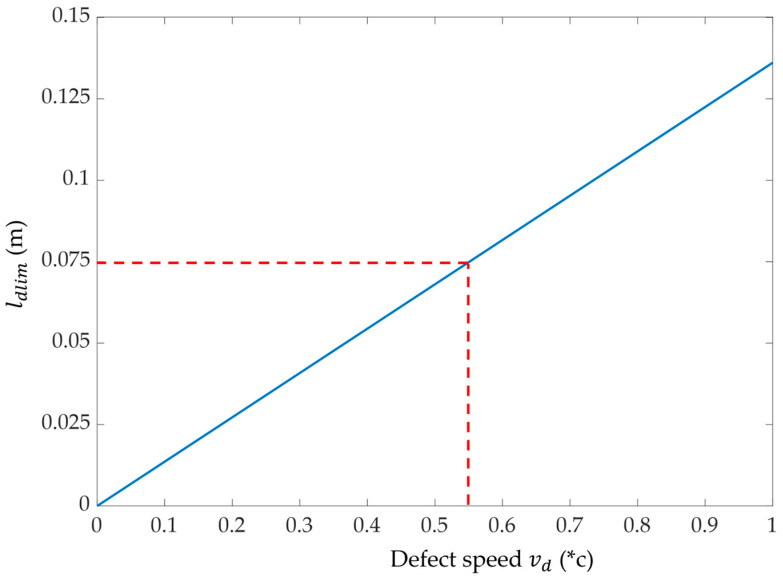
Minimum defect length *l_dlim_* (**-**) under which the amplitude of the overlapping effect will occur, as a function of the defect speed *v_d_* and for *f_c_* = 1102 MHz.

The measured TDR responses are shown in Figure 6, and they are in very good agreement with the model calculated using (8). For a 35 cm defect, the two peaks corresponding to the reflections at the input and output interfaces are well separated, while for shorter defects, the peaks tend to overlap and compensate for each other. This is in agreement with the criterion (9) plotted in Figure 7 which shows that considering $f_{c}=$1102 MHz and a propagation speed inside the defect $v_{d}≅0.55c$, where *c* is the speed of light, the overlapping effect is negligible as long as $l_{d}\geq$ 7.5 cm.

In Figure 6, with a defect length of 12.5 cm close to this limit, the two peaks start to overlap but the overall amplitude is not yet affected. The third reflection, observed experimentally at 0.5 m, is weak but still distinguishable. For the 2.5 cm and 5 cm defects that are shorter than as $l_{dlim}$, the overlapping greatly affects the apparent amplitude of the observed signature. The model deviates slightly because only the first two reflections are taken into account, while in the experimental results, many successive reflections overlap. Nonetheless, this simplified model still pretty accurately describes the experimental signatures and one could include higher-order contributions if more precision was needed.

## 4. Conclusions

The model developed in this paper explains the very peculiar “S” shape of the response produced by a soft defect on a line under test with time-domain reflectometry. The “S” shape is explained as being the result of the overlapping of mainly the first two reflections inside the defect. The physical and electrical parameters influencing this signature were also evidenced and it was also demonstrated that below a minimum defect length, the magnitude of the signature is severely affected by the overlapping echoes. The derived model was experimentally validated using an original defect setup made with microstrips. It was shown that a weak “S”-shaped pattern can be misleadingly interpreted as a small impedance change and consequently one should be extremely careful when analyzing such signatures for diagnostic purposes. Further care must be taken since knowledge of the impedance mismatch alone cannot allow us to reach a conclusion on the severity of a defect. As a matter of fact, a little chafing on a wire would result in a very small impedance mismatch and would still be a very high risk of arcing and potentially catastrophic failure. The new insight developed in this work on the signature of soft defects will benefit the development of new suitable detection methods.

## Data Availability

Data are contained within the article.
